# Encountering nursing home residents’ expressions of death and dying

**DOI:** 10.1177/09697330261465875

**Published:** 2026-07-09

**Authors:** Peter Lundberg, Jane Österlind, Malin Olsson

**Affiliations:** 1 Department of Health Care Sciences, 7643 Marie Cederschiöld University, Stockholm, Sweden

**Keywords:** older adult, nursing home, residential care setting, conversation, death and dying

## Abstract

**Background:**

Encounters with nursing home residents often involve ethically sensitive situations involving fundamental values such as dignity, autonomy, and responding to existential suffering. Their everyday life in nursing homes is shaped by bodily decline, loss of independence, and social isolation, which may evoke reflections on death and dying. Such expressions frequently arise spontaneously in everyday care and may challenge health care professionals’ moral sensitivity, and professional responsibility within organisational constraints. Prior research has explored such expressions but knowledge regarding how health care professionals engage in ethically sensitive conversations remains limited.

**Aim:**

To describe healthcare professionals’ experiences of encountering nursing home residents’ expressions of death and dying.

**Research design:**

A qualitative descriptive study was conducted using semi-structured interviews, which were analysed using inductive qualitative content analysis.

**Participants and research context:**

A total of 12 health care professionals – five assistant nurses, four registered nurses and three physicians – employed or consulting at nursing homes for older people.

**Ethical consideration:**

Ethical aspects were considered, and the study was approved by the Ethical Review Authority in Sweden (Dnr 2022-04026-01).

**Results:**

We identified four themes that emerged from the participants’ response: *Trying to find space and opportunity to talk about death and dying, Striving to understand what lies behind expressions of longing for death, Feeling emotionally attached, Navigating intertwined competences in encounters about death and dying.*

**Conclusion:**

This study shows that conversations about death and dying in nursing homes often arise spontaneously in daily care and rely heavily on trusting relationships between residents in nursing homes and healthcare professionals. Such encounters require emotional presence, moral sensitivity and an ability to witness suffering rather than primarily attempting to solve it.

## Introduction

Healthcare professionals (HCPs) encounter nursing home (NH) residents in everyday interactions where care is intertwined with relational and existential dimensions.^1–3^ These encounters involve moments when NH residents reflect on their lives and on death and dying, placing HCPs in situations that require attentiveness beyond physical care. Spontaneous expressions of a need to talk about death and dying among older persons residing in NHs can pose ethical challenges for HCPs, as they involve fundamental values such as dignity, autonomy, and responses to existential suffering.^3–6^

## Background

Although prior research has addressed ethical challenges in encounters involving death and dying,^7–10^ knowledge regarding how HCPs engage in ethically sensitive conversations with NH residents remains limited. Such conversations require moral sensitivity to the needs of residents in NHs to talk about death and dying in everyday life. How ethically sensitive situations are navigated is shaped not only by HCPs’ moral sensitivity, but also requires an organisation that supports an ethically sensitive care culture.

The ethical challenges can be described as stemming from existential concerns, encompassing fundamental questions about death, freedom, meaninglessness, and isolation.^11^ For NH residents, daily life often involves both meaningful activities and a loss of independence, which may trigger reflections on life’s meaning and their own mortality.^12,13^ Understanding these reflections is crucial for HCPs when responding to NH residents about death and dying in everyday care.

Conversations about death and dying can be proactive and formal, concerning advance care planning (ACP) and the need to make decisions about end-of-life (EOL) issues.^14^ They may also encompass ethical and existential dimensions; however, these aspects are not necessarily explored or made visible within decision-oriented formats. They can also be spontaneous, emerging when a resident expresses reflections on death and dying, sometimes articulated as a longing for death or statements that may be understood as expressions of existential suffering rather than a wish for death per se.^8,15^ Rather than constituting a clear division, formal and spontaneous conversations often overlap, as everyday interactions may open spaces for existential meaning-making beyond administrative or medical agendas. As MacDonald et al.^16^ highlight, spontaneous conversations or ‘small-talk’ can be useful for establishing a connection and may evolve into ‘big talk’ fostering trust and easing sensitive topics.

Various HCPs in NHs encounter residents within their respective roles. In Sweden assistant nurses (ANs) are closest to residents in their everyday care, registered nurses (RNs) oversee nursing and care, and physicians (MDs) are responsible for medical treatment. Despite these different roles, the care provided is inherently interprofessional.^1,17^ The intersections between professional roles are particularly evident in existential conversations, which rarely fall to a single profession and can become particularly complex when spontaneous, ethically challenging issues – such as longing for or hastening death – arise.^18^

For HCPs, caring is about participating in the residents’ daily lives and establishing relationships. It includes respecting routines and preferences, as well as providing care in collaboration with colleagues and other professionals.^19^ Care requires sensitivity to residents’ existential orientations because neglecting them may threaten their autonomy and dignity.^4,20^ This requires moral sensitivity, understood as a personal capacity to attend to and recognise the moral significance of a situation, involving awareness of one’s own role and responsibility, and the ability to distinguish between feelings, facts and values as a prerequisite for moral deliberation.^5^

Previous research shows that NH residents express a need to talk about death and dying in meaningful ways, while HCPs describe challenges in being prepared for and able to engage in such conversations.^7,21^ As encounters often occur in everyday care situations rather than planned settings, they may present ethical challenges for HCPs.

Several studies show that discussing death, dying, or existential issues with NH residents remains a challenge and HCPs may lack the skills or confidence to engage with such sensitive topics.^2,7,9,10^ Alftberg et al.^7^ further highlight that HCPs may also lack the confidence or skills to address existential issues, and time constraints and emotional strain can further hinder engagement. When HCPs recognise what would be an ethically appropriate response but experience organisational or situational constraints that prevent them from acting accordingly, such situations may give rise to moral distress.^6,22^ This underscores the importance of an attentive and ethically supportive leadership.^22^

There is growing interest in how HCPs encounter older persons in relation to existential issues in ethically challenging situations in care.^17,23,24^ Providing existential support in ethically conditioned situations can strengthen older persons’ sense of meaning, contributing to well-being even in the face of decline. Existential support is not confined to a single profession but can be provided by all those involved in care.^25^ Conversely, access to ethical support, interprofessional reflection and shared responsibility may contribute to moral sensitivity. However, knowledge remains limited regarding how HCPs navigate spontaneous ethically challenging conversations about death and dying with NH residents.

## Aim

To describe healthcare professionals’ experiences of encountering nursing home residents’ expressions of death and dying.

## Research design

### Study design

This study employed a qualitative descriptive design using an inductive qualitative content analysis approach.^26^

### Participants, procedure and setting

A purposive sample of 12 HCPs participated in the study (Table 1). The inclusion criteria for participation were more than 3 years’ work experience, more than 1 year at the NH, post-qualification as an assistant nurse (AN) or post-licensure, registered nurse (RN) or physician (MD). The managers of the NHs provided written information to interested employees. Those wishing to participate contacted the first author by e-mail and were subsequently telephoned to confirm participation and arrange the interview time and location. The setting was five NHs in a major Swedish city, each with 36–83 residents. Four NHs had units for both somatic and dementia care; one was exclusively for people with dementia. At all the NHs RNs were on duty during daytime hours (weekdays, and daytime at weekends where applicable), while a team of RNs covered evenings, nights and weekends across multiple sites. ANs staffed the units continuously, day and night. MDs were on site a few hours per week and otherwise available by telephone; the MDs were employed by a specialised medical organisation providing services to several NHs in the county. The first author obtained written informed consent from all the participants before the interviews.

**Table 1.** Description of professionals.

Professional role	(n = 12)	Education
AN	n = 5	High school focusing on caring
MD	n = 3	Specialist geriatric or general competence
RN	n = 4	Specialist older care, palliative care or another field
Age range	32–64 years	​
Experience	4.5–36 years	​

### Data collection

Semi-structured interviews, lasting between 24 and 50 min, were conducted by the first author from March to May 2023, using a formal interview guide and regular follow-up questions.^27^ The interviews were conducted face-to-face at times and locations convenient for the participants. Interview questions covered palliative care and open-ended questions about how HCPs encountered issues of daily and future life, death, dying, and wishes to die as expressed by older persons residing in NHs. In this study we focus on the responses to open-ended questions about older peoples’ expressions of death and dying, as well as their expressed wishes to die. The questions are presented in Table 2.

**Table 2.** Formal interview guide.

Questions
Can you describe an occasion when you spoke with an older person about their perspectives and questions regarding death and dying and the final phase of life. What do you think when an older person express that they wish to die or want help to hasten their death? What do you think your role and task within the team is regarding conversation about end-of life issues?

### Data analysis

We conducted an inductive qualitative content analysis following the approach described by Bengtsson.^26^ This approach comprises four steps^1^: decontextualisation – identifying meaning units^2^; recontextualisation – comparing meaning units with the original text^3^; categorisation – grouping homogeneous units; and^4^ compilation – drawing realistic conclusions. The analysis included both manifest and latent content, focusing on underlying meanings in how HCPs described their encounters with NH residents’ expressions of death and dying. The interviews were transcribed verbatim, after which the transcripts were read while simultaneously listening to the audio recordings to both develop a general understanding and to verify accuracy.

The transcripts were subsequently read through several times to obtain a comprehensive understanding of the data. Meaning units were identified and condensed while preserving their essential content. These units were examined for patterns and relationships and iteratively coded; similar meaning units were grouped into codes, with repeated reference to the original transcripts to ensure accuracy. Codes that shared the same central meaning were grouped together into categories. The authors held continuous discussions throughout the analysis to enhance the trustworthiness and credibility of the thematization.^28^ The analytical process continued until no further abstraction was deemed necessary. Finally, codes and selected quotations were translated into English. An example of the analysis process is presented in Table 3.

**Table 3.** Example of analysis process.

Meaning unit	Condensed meaning unit	Code	Category	Theme
Is this how you think? What is it that you want? Is there something special you wish for, or someone important to you?” there is quite a difference, because many say they no longer want to live due to feeling depressed or sad about their loss of function. But for those who truly wish for death, you might need to respond by asking, how would you like things to be? They may then feel more secure. (8, MD)	Encountering persons who say they want to die or ask for help to die may reflect many different motives. Some are calling for help, feeling anxious or angry, while others have a genuine wish to die. In such situations, one needs to respond by asking how they would like it to be and whether there is something especially important to them. Some may feel reassured by this.	During encounters, persons who wish to die can express different things, but the response involves trying to find out if there is something we can do.	To probe the underlying causes and either address the problem or accept it.	Striving to understand what lies beneath expressions of longing for death
I’ve had several such conversations, but I wish I’d had more. Sometimes you hold back a bit – it has to be the right moment. (6, RN)	Meeting people with a need to talk about existential matters but holding back from having those conversations because one wants the timing to be right.	Talking with people about death should happen at the right moment.	Sensitivity for the right moment	Trying to find space and opportunity to talk about death and dying
I get to know them and see them go from being a bit healthier to becoming more ill, and that really moves me. I work with them every day, so of course I get sad. Some of them leave marks on your heart; you can’t forget them. But at the same time, it’s my role to care, and I can’t show too much emotion. (5, AN)	Getting to know people and seeing them go from being relatively well to becoming more ill creates a deep emotional response, as we have developed a relationship. Sadness must not be shown outwardly, because the role is to provide care.	To know a person is to develop a relationship over time while remaining professional and providing care.	To be close and become part of the person’s life in a relationship, while still providing professional care.	Feeling emotionally attached
In some nursing homes, there are nurses with long experience in older people care and dementia. I think it’s often better that they have those conversations, as they know the residents much better than I do as a doctor – they see them several times a week, while I might meet them once a month at best. (12, MD)	Conversations with residents or relatives about end-of-life care can be carried out by nurses who know the person much better and have a stronger relationship with them than a doctor does, but this presupposes that the nurse has long experience.	Recognising the need to rely on others’ relational competence in conversations about death.	Mutual interdependence through reliance on others’ relational and communicative competence.	Navigating intertwined competences in encounters about death and dying

## Ethical considerations

Both verbal and written informed consent were obtained from all participants. The study was approved by the Swedish Ethics Review Authority (Dnr. 2022-04026-01), and participants were assured of full confidentiality. The results are presented at group level to protect individual identities. The potential benefits of participation were considered to outweigh any risks, as the study provided an opportunity for HCPs to share their experiences and reflect on their thoughts. Participation was voluntary, and the participants were informed that they could withdraw from the study at any time.

## Trustworthiness

Trustworthiness was addressed through credibility, dependability, transferability and confirmability.^28^ Credibility was ensured by careful attention to the analytical process and by seeking agreement among the authors to achieve a shared interpretation of the results. Dependability was supported by situating data interpretation within the broader context of data collection and acknowledging that the researcher’s perspectives evolved through reflection and engagement with the material. For example, the order of the questions was adjusted in later interviews as the researcher’s understanding of the phenomenon deepened. Transferability is primarily judged by the reader but was facilitated through detailed descriptions of the study context, sampling and analytical procedures, and consideration of relevance to other settings. To support confirmability, the results are presented in a way that allows for alternative interpretations. As the first author is an RN who is experienced in the care of older people and well used to the context, the results were discussed within the research group to minimise the influence of preconceptions.

## Results

Data analysis identified four themes: *Trying to find space and opportunity to talk about death and dying, Striving to understand what lies behind expressions of longing for death, Feeling emotionally attached, Navigating intertwined competences in encounters about death and dying.*

### Trying to find space and opportunity to talk about death and dying

This theme describes how HCPs try to find space and opportunities, and preparedness for conversations about death and dying in everyday care. It illustrates how such moments may arise spontaneously during routine interactions and how staff remain attentive to expressions or cues that signal an openness to talk about such issues.

The HCPs described the existence of formal routines for having conversations about dying and death, which range from identifying wishes at the time of admission to the final milestone conversation before death. They emphasised, however, that conversations about death should not be confined to formal routines but should arise when the moment feels right. Opportunities emerged in daily life situations, initiated by either the resident or the HCPs.

When we admit patients here, it’s included as one of the background questions, and it’s almost considered desirable to have that kind of conversation fairly early on…But I think you have to find the right moment and it can take time. (6, RN)

The HCPs described how talking about death and dying purely because it was a requirement could feel strange, particularly in situations where the person did not consider the issue relevant.

As long as they are feeling relatively well, they don’t usually bring it up but it is more that I have an obligation to ask these questions when I admit the patient, and then it can become a bit strange. At first, you sit and talk and have a pleasant conversation, sharing all your plans, and then you suddenly talk about death, but we have to do it. (10, MD)

Instead of waiting for the right moment to occur, HCPs could create the moment or be prepared for conversations in daily care:

I would sort of think that that person would probably be suited to going out for a walk. Or else you would sit in the room and have coffee and… or someone might want… that you fix their hair while you talk. One would sort of try to adapt it… into something that feels good. (11, AN)

For HCPs, conversations about death and dying were about creating possibilities, being attentive, and listening during everyday care. Such conversations could be spontaneously initiated by the resident or by the HCPs when they sensed that the moment was right.

### Striving to understand what lies behind expressions of longing for death

This theme highlights how HCPs encounter and respond to residents’ expressions of longing for death. It describes their efforts to understand what lies behind such expressions by listening, asking questions, and exploring the circumstances shaping the residents’ views.

For HCPs, encounters with residents expressing thoughts about death, or sometimes longing for it, were seen as either an acceptance of a complete life or dissatisfaction with their current circumstances. They expressed wanting to understand the reasoning underlying these views, emphasising listening and understanding, while also wanting to offer alternative perspectives or meaningful goals without neglecting their views. Such expressions were not always interpreted as a wish to hasten death, but sometimes as a longing for death as a form of release or as a need for accompaniment in the process of letting go. One MD described such an encounter as recognising the resident’s sense of agency and acceptance, where the professional role became one of accompanying rather than intervening:

‘…she felt it was a relief to let go…in a way it was quite powerful to see that clear gaze, and that, like, she was fully in control herself somehow… and then it was just for the rest of us to follow along, and walk beside her…’ (10, MD)

When residents expressed a wish to die, receive assistance in dying, or hasten death, HCPs explored the reasons and the arguments presented. For instance, treating depressive symptoms, when possible, while accepting that some residents viewed their life as complete. With some residents attempts might be made to redirect the focus through distraction or meaningful activities, as illustrated below:

A resident who often says, what is the point, I want to die. Then it can become more of a conversation about what it is that makes them not want to live any longer. And then they say, there’s no point. It’s boring here. I am so ill. I don’t want to be around anymore… you don’t think you would miss anything, is there anything that keeps you here… (11, AN)

The HCPs described approaching the resident with curiosity, openness and fearlessness, while allowing them to express their feelings freely. They tried to understand, sometimes seeking solutions through medication or distraction, sometimes listening and following the residents’ views in acceptance.

### Feeling emotionally attached

This theme describes how close relationships between HCPs and residents in NHs create emotional connections that support trust but may also lead to emotional strain. It illustrates how staff experience the need to balance emotional closeness with maintaining an appropriate professional distance.

Those I care for… I have become very close to many of them… so it is… very much… I mean, it feels like that, that it is difficult, you know… you always have to be attentive and make them feel safe, that I am there… don’t be afraid. (2, AN)

The HCPs expressed powerlessness and uncertainty when residents expressed a wish to die, wanting to respond without harm but struggling when convictions seemed firm. Sometimes the HCPs interpreted the residents’ expressions as ambivalent.

… it is difficult to know whether it is completely serious, because it can come quite spontaneously, that is, I, can’t I just move on, can’t I just die now. Now it’s enough, you know. (3, AN)

The HCPs described that relationship-building was essential for conversations about death and dying, although the depth of relationships varied by professional role. However, they also expressed uncertainty about how to respond appropriately without causing harm.

### Navigating intertwined competences in encounters about death and dying

This theme describes how HCPs navigate the interplay of different professional roles and competences in relation to their encounters with NH residents. It captures how they experience and manage their professional limitations when responding to residents’ existential expressions, while simultaneously relying on other team members’ relational and communicative competences. The theme highlights how encounters with death and dying are understood as complex, with shared responsibilities that extend beyond medical treatment and which require interprofessional collaboration to meet the NH residents’ needs.

One MD described their dependence on other HCPs and their own limitations as medical professionals in the teamwork.

I do believe that it’s the nursing staff who do the most important work here. I can prescribe some medication for symptoms, but I work at several different care homes so I don’t have time to see everyone every week. It’s about relieving symptoms as best as possible, and then the nursing staff are there, supporting in the ways they can. (12, MD)

HCPs described how encounters with residents’ existential expressions often highlighted their own professional limitations. Rather than having answers to questions about death, they emphasised the importance of recognising uncertainty and accepting that some needs lay beyond their competence. Acknowledging these limitations was described as an essential part of providing care, where presence and openness could be more meaningful than attempts to explain or resolve existential concerns:

Sometimes I think that it may involve a certain degree of uncertainty. The patient says, ‘I need to talk to someone’. But sometimes it may be that they would like to talk to a priest or similar… and that the conversations quite often involve being able to sit in silence… you can get quite far with that… I don’t have many answers about death either. (6, RN)

Conversations about death were not always described as explicit verbal exchanges with residents but can instead be understood as embedded in relational practices surrounding death. Managing death and creating space for reflection were interpreted as expressions of engagement in death that extends beyond medical treatment and conventional dialogue.

I can’t help with that [to die], but I can be here for you—helping you feel safe and offering my presence and support. (2, AN)

Understanding that encounters with death are complex and beyond medical treatment is illustrated by an AN:

And then I have really tried to push for this, which is very, very difficult - that we, as staff, of course should have the opportunity to come together. At least for five or ten minutes… and acknowledge it.(11, AN)

The HCPs’ description of their encounters with residents show that death is understood as more than a medical event, as encounters with death and dying are described as giving rise to emotional and existential needs among HCPs. Understanding death as complex involves not only managing the physical aspects of dying but also creating space to talk about death with residents and colleagues. At the same time, HCPs described organisational constraints as limiting opportunities for such conversations.

## Discussion

In this study, we aimed to describe HCPs’ experiences of encountering NH residents’ expressions of death and dying. The results show that HCPs seek opportunities to discuss such issues. They also try to understand what lies behind residents’ expressions of longing for death. The HCPs expressed how they become emotionally attached to residents, which requires balancing closeness and professional distance. Mutual interdependence between HCPs was considered essential for supporting NH residents in expressing their views. Our analysis highlights the need for an ethically sensitive organisation that supports HCPs in engaging in spontaneous and often delicate existential conversations.

The results have been interpreted after consideration of prior research focusing on ethically sensitive organisations.^22^ Further, our results are discussed in relation to research concerning the needs among NH residents to experience dignity in daily life, death and dying,^4,29^ theory about the importance of relationships based on a balance between closeness and distance,^30^ and the practice of witnessing.^31^

The present study also highlights how mutual interdependence between HCPs, emotional strain, and the strategies used when encountering NH residents’ expressions about death and dying influence the ability of HCPs to maintain moral sensitivity. Our results confirm earlier research showing that spontaneous conversations about death and dying can feel more natural and meaningful than formalised ones.^8^ However, while previous studies mainly describe spontaneous conversations as a phenomenon, our results clarify *how* HCPs actively create space for spontaneity. The HCPs emphasise preparedness and relationship as prerequisites for recognising ‘the right moment’. This highlights spontaneity as not being merely accidental but relationally cultivated. Creating space for spontaneous conversations in everyday care may contribute to maintaining the residents’ dignity by avoiding the reduction of the unique individual’s status to that of a patient. Dignity is achieved where a mutual and respectful encounter arises. When HCPs are prepared for such encounters, this may also help prevent them from experiencing moral distress. However, formal and spontaneous conversations should not be considered mutually exclusive but instead as complementary. The discomfort some HCPs expressed in relation to formalised conversations about death and dying should not be interpreted as resistance to ethical reflection. Rather, it reflects a moral sensitivity to relational timing and the risk that pre-defined formats may disrupt trustful encounters when existential issues are present.

Whether they are formal or spontaneous, conversations about death and dying should always be approached with moral sensitivity. This can be illustrated by the metaphor of the relationship with the resident as being like a bridge, a means of connecting with them, establishing closeness, yet also maintaining distance and respect.^30^ The tension of closeness and distance is highlighted by Bäckersten et al.^25^ who describe various approaches to issues concerning death and dying. While some persons appreciate a trustful relationship when talking about these issues, others prefer to talk to professionals such as nurses or physicians. Hence, the trustful relationship with the HCPs is crucial for successful conversations.

According to Fantus et al.,^22^ moral distress can be reduced through interprofessional reflection, mutual understanding of roles, ethical leadership, and participation in policy development. The results in our study imply that organisations have a responsibility to foster an ethically sensitive culture within relationship-based care. The HCPs’ have the ability to balance closeness and professional distance, to witness rather than solve, and to create space for spontaneous conversation. Organisations should arrange education in talking about death and dying to foster security and create a care culture that includes both possibilities and space in daily care for such conversations. They should also introduce interprofessional forums for ethical discussions. This would support ethically focused care based on respect for the residents’ dignity and promote HCPs moral sensitivity.^32^

The HCPs described encountering NH residents’ views on death and dying as situations where they might either accept these views, feel frustrated, or attempt to distract or help the person reframe the situation and identify new goals. These results correspond with Alftberg et al.^7^ who claimed that personnel distract, comfort or disregard the NH residents in these encounters. Our results highlight that there is a tension between empathy and the risk of becoming patronising which may undermine autonomy and dignity. Moral sensitivity is therefore needed to balance problem-solving and listening. Within a relationship grounded in trust, as highlighted by Kang et al.,^33^ NH residents are more likely to choose when and how to talk about death and dying shifting the HCPs role towards listening rather than problem-solving. When HCPs are prepared, they can let such conversations unfold naturally. This may divert the focus away from problem-solving towards witnessing, which in turn may reduce moral distress. Witnessing involves being present and recognising another person´s suffering, rather than trying to remove it.^31^ The focus is on *how* they feel rather than *why* they feel. While HCPs described how to understand and sometimes alleviate residents’ expressions through action, the notion of witnessing highlights a complementary stance – one of being present without the need to solve, allowing suffering to be shared rather than removed. Witnessing therefore complements problem-solving by engaging the older persons’ life world and experience of suffering. In this context, moral distress does not arise from talking about death as a natural life stage per se, but from the moral tension inherent in these encounters. HCPs may recognise an ethical obligation to acknowledge suffering and respect autonomy, while organisational or normative constraints limit their ability to act accordingly.

There is a need for organisations to prioritise witnessing and provide existential supervision and reflection meetings to cultivate an ethically sensitive care culture. It should be acknowledged as an integral part of care, which is a strategy emphasised by Sundström et al.^3^ Such support legitimises emotion as part of professional ethical practice and strengthens lifeworld-oriented understanding and structured reflection.

Our results indicate that the relationship between HCPs and NH residents is central to trustful care. This aligns with Sjöberg et al.^34^ who have shown that older people experience relief from existential loneliness when they feel seen and acknowledged by care staff, fostering a sense of meaningful togetherness. Such relationships support autonomy by allowing care to be grounded in the older person’s lifeworld. However, close relationships may also create emotional strain and increase the risk of moral distress among HCPs. Alftberg et al.^7^ highlight that when HCPs lack the tools to balance emotional closeness and professionalism, they may interpret views on death as pathological. Our results underline the need to navigate this tension.

Being emotionally attached can support trust but may also generate powerlessness and moral distress. The HCPs must protect themselves from such distress while maintaining empathy. Organisations have a responsibility to acknowledge the tension the HCPs can feel between maintaining empathy and preserving emotional distance when appropriate. Addressing this tension is essential for the provision of ethically sustainable care. An ethically sensitive organisation should be proactive and hold regular ethical forums and provide supervision. This can help decrease moral distress and sustain moral sensitivity.^22^ The HCPs do not want to cause harm yet attempts to avoid harm may become patronising if distraction undermines dignity. Witnessing can connect two life worlds, but organisational support is required to help HCPs manage the reactions and emotions expressed in our study. As shown by Lagerin et al.^18^ the focus of HCPs often prioritises problem-solving over witnessing, yet existential conversations become meaningful when grounded in trust. However, a tension can arise between professionalism and the degree of emotional closeness a caregiver can allow, which Alftberg et al.^7^ emphasise as a central challenge. How close one can be and how much of oneself can be shared are ethical issues; HCPs need to remain emotionally protected while still sustaining connection. This tension can be illustrated by the metaphor of a bridge, balancing closeness and distance.^30^

In this study, we emphasise the need to navigate intertwined competence through mutual interdependence between HCPs and relational communication, acknowledging professional limitations as an ethically inherent condition rather than an individual shortcoming. Such navigation has been shown to reduce ethical burden and strengthen the capacity of HCPs in their encounters with older persons.^22,32,35^ Our results extend beyond previous results by clarifying the tension between medical and relational competence, highlighting how mutual trust enables HCPs to seek and offer support to ensure that the resident receives the most appropriate care. Such an approach can contribute to a cohesive and ethically sustainable practice, grounded in a holistic understanding of the older person.

An ethically sensitive approach to competence may be understood through a synthesis of the bridge metaphor, balancing closeness and distance, together with witnessing. Further, an existential approach positions conversations about death and dying as part of the individual’s ongoing identity formation, in line with Blackie et al.^29^ Such a broadened view of competence supports autonomy and dignity and may reduce moral distress. Organisations should therefore recognise the relationship-based competence of all HCPs and provide regular ethical forums and supervision to foster ethically sensitive care.

### Integrated summary of results and ethical aspects

Taken together, our results show that existential care in NHs can be understood through a relational–existential ethical care framework in which three elements are intertwined^1^: relational balance – managing closeness and distance^2^ witnessing – being present during suffering without prematurely seeking solutions; and^3^ existential identity – recognising conversations about death and dying as part of ongoing identity formation. When these elements are supported by mutual interdependence and an ethically sensitive organisational culture, HCPs are better able to respond to spontaneous existential expressions by NH residents in daily care. Such encounters may strengthen dignity, autonomy and meaning for residents, while reducing moral distress among HCPs. Thus, ethical existential care emerges as both an everyday relational practice and a commitment requiring organisational support.

### Study strengths and limitations

This study has several limitations that should be considered when interpreting the results. The sample included 12 HCPs from five NHs in one Swedish city, which may limit transferability to other contexts. Although different professional groups were represented, participating physicians worked mainly as consultants with limited presence in daily care, which may have influenced the perspectives captured and resulted in a stronger emphasis on nursing staff experiences.

The NHs included both somatic and dementia care units. While many NH residents can engage in conversations about death and dying, the results do not fully reflect encounters involving persons with more advanced cognitive impairment. In addition, the study is based on self-reported interview data, which may be influenced by recall bias and social desirability. Finally, as the interviews were conducted in Swedish and later translated into English, some linguistic and contextual nuances may have been lost despite careful translation.

## Conclusion

This study shows that conversations about death and dying in NHs can arise spontaneously in daily care and rely heavily on trusting relationships between NH residents and HCPs. Such encounters require emotional presence, moral sensitivity and a capacity for witnessing, which involves being present during suffering rather than primarily attempting to solve it. The results also highlight the ethical and emotional challenges this may entail for HCPs and the importance of mutual interdependence between HCPs and supportive organisational conditions that enable time and preparedness for existential care. Strengthening these relational, ethical and organisational dimensions may contribute to more meaningful encounters, promote dignity, and support NH residents’ identity and sense of meaning at the end of life.
